# A Genetic Investigation of Island Jersey Cattle, the Foundation of the Jersey Breed: Comparing Population Structure and Selection to Guernsey, Holstein, and United States Jersey Cattle

**DOI:** 10.3389/fgene.2020.00366

**Published:** 2020-04-17

**Authors:** Heather J. Huson, Tad S. Sonstegard, James Godfrey, David Hambrook, Cari Wolfe, George Wiggans, Harvey Blackburn, Curtis P. VanTassell

**Affiliations:** ^1^Department of Animal Science, Cornell University, Ithaca, NY, United States; ^2^Acceligen, Eagan, MN, United States; ^3^Royal Jersey Agricultural & Horticultural Society, Trinity, Jersey, United Kingdom; ^4^American Jersey Cattle Association, Reynoldsburg, OH, United States; ^5^Council on Dairy Cattle Breeding, Bowie, MD, United States; ^6^National Animal Germplasm Program, USDA-ARS, Fort Collins, CO, United States; ^7^Animal Genomics and Improvement Laboratory, USDA-ARS, Beltsville, MD, United States

**Keywords:** runs of homozygosity, F_ST_, signatures of selection, dairy cattle, Jersey, Jersey Island

## Abstract

For two centuries, Jersey cattle were exported globally, adapting to varying climates and production systems, yet the founding population remained genetically isolated on the Island of Jersey. The Island of Jersey formally allowed the importation of pure Jersey cattle in 2008. This study characterized the genetic variation of 49 popular bulls from the Island of Jersey born from 1964 to 2004 and compared them to 47 non-Island Jersey bulls and cows, primarily from the United States In addition, 21 Guernsey cattle derived from the Island of Guernsey and 71 Holstein cattle served as reference populations for genetic comparison. Cattle were genotyped on the Illumina BovineHD Beadchip producing 777,962 SNPs spanning the genome. Principal component analysis revealed population stratification within breed reflective of individual animal’s continental origin. When compared to Holstein and Guernsey, all Jersey clustered together by breed. The Jersey breed demonstrated increased inbreeding in comparison to Holstein or Guernsey with slightly higher estimates of inbreeding coefficients and identity-by-descent. The Island and United States Jersey have relatively similar, yet statistically different inbreeding estimates despite vastly different population sizes and gene flow. Signatures of selection within Island Jersey were identified using genome-wide homozygosity association and marker-based F_ST_ that provided population informative single-nucleotide polymorphism (SNPs). Biological significance of the homozygosity association results identified multiple genes on chromosomes 5, 24, and 27, involved in immune function and cellular processes. Overall, genomic variation was identified between the Island and non-Island Jersey cattle producing population informative SNPs and differing runs of homozygosity (ROH) over immune regulation and metabolic genes. Results on inbreeding measures and ROH may reflect varying effective population size or differential selection with grazing systems promoting natural selection for traits such as parasite resistance, whereas confinement systems demonstrate a more intensive artificial selection. More broadly, differences in breed formation, particularly between the two Channel Island breeds, likely contributed to the variation in ROH and inbreeding. This research provides a reference for the Jersey breed based on the genetic foundation of the Island cattle as compared to the intensively selected United States cattle, and identifies regions of the genome for future investigation of immune regulation and metabolic processes.

## Introduction

The Jersey cattle breed originated on the Island of Jersey over 200 years ago. Jersey is the southern-most island in the English Channel, off the coast of France. The Jersey breed is one of the oldest dairy breeds with reports from as early as 1771 stating that these cattle were a chief product of the Island. Laws forbidding the importation of livestock and related products to the Island of Jersey were enacted as early as 1763, and these laws were followed with a series of regulations through the next two centuries completely isolating these cattle, initiating the breed formation of the Jersey. By the early 1800’s, the Jersey cow was “…celebrated not only for its beauty, but for the richness in milk and excellence in butter.” – George S. Syvret (1832) (Becker, 1973). The Royal Jersey Agricultural and Horticultural Society (RJA and HS) was formed in 1833 as a means of improving farms, gardening, and, especially, the Island’s cattle. To this end, foundation stocks of Jersey cattle were identified, and the Jersey Herd Book was established on March 3, 1866 with the first inspections of stock undertaken on April 11, 1866. Ancestry of all registered Jersey cattle can be traced back to the Island’s herd book, making it a complete census of the population. While importation of dairy cattle was disallowed entry onto the Island, Jersey cattle were in high demand for export, with 7,330 head shipped to England, America, Australia, France, and New Zealand from 1873 to 1879. These exports continued through the 21st century (Becker, 1973). All cattle on the Island continued to be registered in the RJA and HS herd book, ensuring breed purity. The population was maintained as a closed breeding population until 2008.

Recognizing the performance gap between the populations of Jerseys on the Island and internationally traded Jersey genetics, Jersey Island breeders proposed opening the border to Jersey cattle populations maintained in other countries. After 219 years of isolation, requiring an act of its parliament, the Island of Jersey opened its doors to genetic importation in 2008 (RJA and HS, 2014; JerseyCanada, 2019). This controversial decision was precipitated by depressed productivity and economic viability of Island Jerseys as compared to international Jersey populations. Cryopreserved semen from over 450 Island of Jersey bulls born from the 1960s to present are stored at both the RJA and HS on the Island of Jersey and the National Animal Germplasm Program (NAGP) facility located at the National Center for Genetic Resources Preservation, a part of the USDA’s Agricultural Research Service (RJA and HS, 2014). Importing semen allowed Island farmers to breed their cows to many different bulls having diverse lineages and high genetic merit to close their productivity gap with international competitors. The ability to cryopreserve the current and historic germplasm provided the security and flexibility to revert to the pre-importation genetic composition of the Island Jersey population, if desired. Currently, two out of three calves born on the Island of Jersey are now sired by top international Jersey bulls. A few of the Island farmers are voluntarily closing their herds and not breeding with bulls having non-Island ancestry. These herds will likely use international genetics over time. Early monitoring of the new generation of internationally sired animals has shown improved milk production, conformation, and health traits (RJA and HS, 2014).

Jersey cattle are now found in at least 82 countries around the world, demonstrating their adaptability to a wide range of climatic and geographical conditions. The breed has a relatively small frame with an average weight of 410 kg (900 lb) and produces more milk per unit of body weight than any dairy breed (Oklahoma State University Board of Regents, 2008). Jersey introduction into the United States has focused on selection of animals for milk and butterfat production under intensive commercial farming systems while Island of Jersey cattle are reared in smaller, pasture-based farming systems. There are 24 farms on the Island with an average herd size of 122 cows with approximately 3,000 total milking cows (5,195 total animals; October 2013) (RJA and HS, 2014). In contrast, in 2014 the United States had approximately 272,000 milking Jersey cows, with an average herd size of 290 cows (CDCB, 2015). Island Jerseys produce an average of 5,043 kg of milk per cow and United States Jerseys produce 8,150 kg (CDCB, 2015). Overall, Jersey cows excel with 18% longer productive life than other dairy breeds. Jerseys also possess increased reproductive efficiency when compared to Holsteins for traits including calving interval, days open, age at first calving, and calves per lifetime (U.S. Jersey, 2014).

Here, we compared the genetic diversity of the Island of Jersey cattle to non-Island registered Jersey cattle, particularly those from the United States. Holstein and Guernsey cattle, both dairy breeds, provided a point of reference for inbreeding measures and population structure. In addition, Guernsey cattle are historically similar to Jersey cattle, with that breed originating on the Island of Guernsey, another English Channel island relatively close to the Island of Jersey. Like the Island of Jersey, the Island of Guernsey banned all cattle and germplasm importation from 1877 to 1976. However, competing herd books and less stringent early controls of cattle movement resulted in a degree of leniency in defining Guernsey breed purity (Luff, 2004; RGA and HS, 2016). Using this dataset, our research aimed to (1) identify the extent of population stratification within the Jersey breed considering geographic origin of an animal, (2) detect runs of homozygosity (ROH) and allele frequency variation differentiating the Island Jersey cattle, and (3) to determine inbreeding levels and the frequency of the JH1 mutation of the Island Jersey cattle. Overall, this research provides a reference for the Jersey breed based on the genetic foundation of the Island Jersey cattle. It identifies regions of the genome for future investigation of immune regulation and metabolic processes potentially affecting health and production traits important for management decisions on the Island of Jersey including germplasm import or export regulations and breed conservation.

## Materials and Methods

### Sample Collection and Genotyping

A total of 49 Island Jersey bulls were chosen from the USDA-ARS-NAGP repository for genetic analysis based on their year of birth and pedigree analysis. The cross-section of bulls targeted all available decades of animals spanning from the 1960s to 2000s with germplasm available and those least related to one another (Supplementary Table S1). Parent-offspring and full- or half-sibling relationships were avoided. All other samples were part of the USDA-ARS Animal Genetics and Improvement Laboratory (AGIL) genotype database, the Council on Dairy Cattle Breeding genotyping archive, or the Bovine Genome Database (Bovine Genome et al., 2009; Bovine HapMap et al., 2009). A total of 130 individuals representing United States Jersey (JE_USA; *n* = 38), United States Holstein (HO; *n* = 71), and British Guernsey (GU; *n* = 21) were available for analysis, most having birth year information. The United States Jersey and Holstein populations included 4 and 5 cows, respectively with the majority of the animals being bulls with birthdates spanning each decade starting from 1950. The Guernsey genotypes were obtained from the Bovine HapMap project (Bovine HapMap et al., 2009) and included 20 cows, a single bull, and only three animals with known birth year. Genotypes for Jerseys with registration representing Canada (*n* = 2), New Zealand (*n* = 3), and Denmark (*n* = 3) were available, but when the pedigrees of these animals along with the Jerseys registered in the United States were examined, the country of registry seemed to be less informative than the contribution to each animal from historic pedigree information. With a primary goal of accurately reflecting the origins of the Jersey cattle in this study, pedigree data was used to assign country of origin. The country of registry of the 16 great-great-grandparents (GGGP) were used to assign non-Island Jersey cattle if at least 4 GGGP were from outside the United States Using this approach, the individuals were assigned country representation as follows; Canada (JE_CAN; *n* = 1), New Zealand (JE_NZL; *n* = 3), and Denmark (JE_DNK; *n* = 8). Most notably, this process reassigned a Canadian registered Jersey with all 16 GGGP from the United States as a United States Jersey and 5 United States registered Jersey that had at least 4 GGGP from Denmark as Danish. Unfortunately, having so few data on animals for these non-United States countries, the power of inference was low. The data from these animals were only used for the principal component analyses (PCA). The set of all 95 Jerseys will be noted simply as JE. All non-Island Jersey were initially assigned to a country of origin or population based on the country in which they were registered when genotyped, but several animals were reassigned to different countries based on pedigree evidence (Supplementary Table S2).

Genomic DNA was isolated from semen from the 49 Island Jersey bulls using the QIAGEN Gentra Puregene kit and following standard proteinase K and phenol extraction methods (QIAGEN, 2015). These animals were genotyped using the Illumina BovineHD Beadchip producing 777,962 single-nucleotide polymorphism (SNPs) markers that were called and clustered using the Illumina Genome Studio software (Illumina, 2012). The remaining animals also genotyped using the BovineHD chip, and these data were obtained from the Bovine HapMap consortium (Bovine HapMap et al., 2009) and integrated with the data collected for the Island bulls. Quality control (QC) measures were calculated using Golden Helix SNP and Variation Suite (SVS) (Golden_Helix, 2012) on the combined dataset, and data retained after QC included 636,099 markers having >95% genotyping call rate and >5% minor allele frequency. Markers were also excluded if they were unmapped to the UMD 3.1 bovine genome assembly (*n* = 832) or mapped to sex chromosomes (*n* = 15,629). Sample inclusion required a genotyping call rate of >95% and a pairwise identity-by-descent calculation of <0.75, providing JE_ISL (*n* = 49), JE_USA (*n* = 34), JE_CAN (*n* = 1), JE_NZL (*n* = 3), JE_DNK (*n* = 8), HO (*n* = 65), and GU (*n* = 21) with 619,638 markers spanning the genome for analyses.

### JH1 Fertility Genotype

The JH1 recessive fertility genotype was investigated in the Island Jerseys. The purpose of this investigation was to characterize the presence of the JH1 mutation within the closed population and if present, the frequency. DNA from the 49 Island Jerseys was analyzed for the likely causative nonsense mutation in *CWC15*, by Geneseek using the SEQUENOM iPLEX Gold protocol previously developed (Sonstegard et al., 2013). This application was specific to the single JH1 marker which was assayed in both 5′ and 3′ directions using the following amplification primer sequences of 5′-ACGTTGGATGCTTTAGACAGACCA CTCAGG-3′ and 5′-ACGTTGGATGTCCAACTCTCTCCTGAA GTC-3′and extension primer sequences of 5′-GCCCCTGA AGAGGTT-3′ and 5′-CCTGAAGTCACGGTTTC-3′.

### Y-Chromosome Lineages

To determine Y-chromosome lineages in the Island Jersey animals, the nine hemizygous SNP markers present on both the BovineHD and Bovine LD (Boichard et al., 2012) genotyping assays were used to determine haplotypes. Marker genotypes were exported as text files from the Illumina GenomeStudio [11], and resulting haplotypes were compared with those generated from genotypes for males from the Bovine HapMap population (Bovine HapMap et al., 2009).

### Principal Component Analysis

Principal component analysis (PCA) was used to investigate population structure within the Jersey breed and to compare Jersey, Holstein, and Guernsey breeds using Golden Helix SVS software (Golden_Helix, 2012). Analyses were conducted with an additive model identifying 10 principal components with markers normalized by their theoretical standard deviation under Hardy-Weinberg equilibrium. In total, four different datasets were analyzed by PCA to compare across all three breeds, between the two Channel Island breeds, and within the Jersey breed (Supplementary Table S2: Sample # after QC). The first PCA compared all 181 cattle represented as Jersey (JE; *n* = 95), Holstein (HO; *n* = 65), and Guernsey (GU; *n* = 21). The second PCA compared only the Jersey (JE; *n* = 95) and Guernsey (GU; *n* = 21) breeds that originated on nearby English Channel Islands. An additional PCA compared the Jersey (JE; *n* = 95) and Holstein (HO; *n* = 65) breeds. Lastly, Jersey (JE; *n* = 95) cattle were analyzed independently to characterize sub-structure within the breed. All PCA were completed using the data for 619,638 autosomal SNPs identified after QC edits.

### Admixture Analysis

Breed purity, focusing primarily on the Jersey breed, was analyzed using the software ADMIXTURE: fast ancestry estimation version 1.3.0 (Alexander et al., 2009; Alexander and Lange, 2011). Data input files were generated for ADMIXTURE using PLINK software version 1.07 (Purcell et al., 2007). The same dataset including 181 individuals representing all three breeds genotyped for the same 619,638 SNPs was used for both PCA and ADMIXTURE investigations allowing comparison of population structure across analyses. Using unsupervised clustering analysis, the dataset was analyzed with *K* = 2, 3, 4, and 5, where *K* represents the number of genetic clusters or populations. Cross-validation error values were used to determine the *K* value with the best predictive accuracy. These populations are based upon allele frequencies of the inferred ancestral population. Each analysis was replicated five times to assess uniformity of results. The average of the five replicates for each *K* value are presented. These averages are based on the entire dataset of 181 individuals. Recognizing that the imbalanced number of individuals representing the different populations could bias these analyses, additional evaluations of *K* = 2, 3, and 4 using a dataset of 60 individuals comprised of 20 individuals representing each breed was conducted. The Jersey breed included ten randomly selected individuals from each of the Island and United States populations. Cross-validation (CV) procedures were used to identify the optimal number of genetic populations, *K*-value, for each dataset.

### Signatures of Selection

Signatures of genetic selection associated to the Island Jersey were investigated using marker-based F_ST_. This analysis generates an F_ST_ value for each individual SNP, comparing the sub-populations of Island Jersey to non-Island Jersey. A genome-wide analysis of 619,638 autosomal SNPs was conducted using Golden Helix SVS software (Golden_Helix, 2012). Forty-nine Jersey Island bulls were compared to 46 non-Island Jersey (42 bulls; 4 cows) to assess variation in F_ST_ marker analysis. The same 49 Island Jersey were compared to the 38 United States Jersey, the only other Jersey population having a comparable number of individuals.

### Runs of Homozygosity

An assessment of the ROH was conducted for identification of conserved genomic regions common among all Jersey populations and those different between the Island Jersey and non-Island Jersey. An ROH is defined as a region of the genome where consecutive genetic markers are uniformly homozygous. Similar to the marker-based F_ST_ analysis, Island Jerseys were compared to all non-Island Jerseys, and a separate analysis of Island Jerseys compared to the United States Jerseys. Golden Helix SVS software was used to assess 619,638 SNPs with the following parameters: the minimum length of an ROH was 500 kilobasepair (Kb), a minimum of 25 SNPs within an ROH, one heterozygote allowed, missing genotypes at a maximum of 5 loci, and maximum gap between consecutive SNPs of 100 Kb (Golden_Helix, 2016). These thresholds were selected based on the use of the BovineHD beadchip with over 10-fold increase in markers genotyped than studies using the BovineSNP50. The thresholds used here were similar to those used by Purfield et al. (2012) using the same genotyping assay. Despite the strict definition of a ROH being composed of all homozygous markers, a single heterozygous position was permitted to accommodate potential genotyping errors. Several ROH data outputs were generated, including specific ROH for each animal analyzed, clusters of ROH found common within an analysis, and the incidence of SNPs occurring in ROH (Golden_Helix, 2016). From these outputs, the following details were calculated and a genome-wide homozygosity association test based on ROH was conducted.

First, ROH were identified for each animal (*n* = 173, excluding non-Island or United States Jersey). Then, clusters were identified across all individuals. A cluster was identified as a common region where at least five individuals had a ROH, while each individual may have unique start and end points for their specific ROH. The cluster is identified as the consensus ROH found in those five or more individuals – this consensus is the region from the maximum of the starting ROH positions to the minimum of the end positions.

To adjust for different numbers of animals represented within each breed, calculations were simplified to the average number or size of ROH per individual within a breed for further comparisons. The total number of ROH were calculated and reduced to the number of ROH observed for a given length of the run. The lengths of the ROH were binned in megabasepairs (Mb) as follows: >0.5; >2; >4; >8; >16 Mb. The number of times a SNP was included in an ROH was used to identify the most common ROH. Lastly, regions identified in the clusters of runs were used to denote start and end points pertaining to the ROH genome-wide association (GWA) results.

A numeric association test was conducted using an established whole genome homozygosity association method developed by Lencz et al. (2007). This approach was developed in collaboration with Golden Helix and is now available in the SVS software (Golden_Helix, 2016). This analysis uses the Golden Helix output “*First column of each cluster*” that is a calculation of the proportion of SNPs in each cluster that are members of common ROH. By only using the first SNP of each established ROH cluster (91,754 SNPs) the total number of tests is reduced for Bonferroni multiple testing correction. Island Jersey were compared to either non-Island (including United States, DNK, NZL, CAN) or United States Jersey and were the dependent variable in the association analysis. The purpose of this test was to identify conserved homozygous regions of the genome associated with either the Island or non-Island Jersey.

### Gene Pathway Analysis

Regions identified by the marker-based F_ST_ and ROH analyses were examined for genes of biological significance using PANTHER software version 10.0 (Mi et al., 2013a, b). Lists of genes located in regions specific to the F_ST_ and ROH results were identified using the UMD 3.1 bovine genome assembly annotation in the UCSC Genome Browser and confirmed with the updated UMD 3.1.1 assembly (UCSC, 2013). These lists of genes were submitted to PANTHER to determine over- and under-represented biological processes among the genes in the given cluster. F_ST_ regions were identified by 31 SNPs having the highest F_ST_ score. These SNP exceeded seven standard deviations, i.e., F_ST_ > 7$\hat{\sigma }$ ($\hat{\sigma }$ = 0.084; 7$\hat{\sigma }$ = 0.641) above the mean ($\hat{\mu }$ = 0.056) F_ST_. The region surrounding each of the 31 SNPs was extended depending on the F_ST_ values of the neighboring SNPs. If the F_ST_ of both of the SNPs flanking the extreme SNP were a maximum of five standard deviations F_ST_ ≤ 5$\hat{\sigma }$ (5$\hat{\sigma }$ = 0.474) above the mean, then a single Mb region was specified with that extreme SNP forming the center of that region. Otherwise, starting at the extreme SNP, the region was extended by adding SNPs in each direction while F_ST_ $>\hat{5\,\sigma }$ for each SNP, and an additional Mb was added at each end of the contiguous segment of SNPs with all F_ST_ > 5$\hat{\sigma }$ and at least one SNP with F_ST_ > 7$\hat{\sigma }$. The maximum number of consecutive SNPs within a region was six and found at multiple locations, and the longest distance between the start and end SNPs was 38.5 Kb. Regions were investigated individually for annotated genes and subjected to PANTHER gene pathway analysis. In addition, genes from all identified regions were combined and studied with PANTHER gene pathway analysis to provide a broader overview for gene selection occurring across the genome.

Common ROH regions among all three breeds and also within Jersey cattle were identified using an approach similar to the method used to select the F_ST_ regions. First, select SNPs observed in an ROH, and then identify the SNPs that reached a threshold of seven standard deviations above the mean number of times SNPs occurred in an ROH. The region was recognized by consecutive SNPs reaching greater than five standard deviations above the mean number of observances. Lastly, biological variation between Island and non-Island Jerseys was investigated using the homozygosity association test results. In this method, ROH regions with a *p*-value less than or equal to 0.001 from the numeric association test were analyzed in the PANTHER gene pathway system. The endpoints of the ROH were identified by Golden Helix SVS software in the clusters of runs output used for the association testing. Like F_ST_, genes within each region were collated and investigated per region and then in a combined analysis of all regions.

### Inbreeding Estimations

Identity-by-descent (IBD) and F-statistics were generated to assess individual inbreeding, pair-wise comparison of relatedness, and population divergence. Inbreeding measures including IBD and Wright’s F-statistics were calculated in Golden Helix SVS using 129,336 SNPs spanning the genome after linkage disequilibrium (LD) pruning. Filtering for LD used the following parameters of window size equal to 50 SNPs with a frameshift of 5 SNP increments and *r*^2^ greater than or equal to 0.5. IBD estimates the probability that the alleles of two individuals came from the same ancestral chromosome. Hence, a pairwise comparison of every individual within each breed was generated providing an IBD estimate for each pair. The IBD calculation first determines the identity-by-state (IBS) between pairs of individuals, which reflects whether the individuals share 0, 1, or 2 alleles at each SNP. IBS calculations are then used to estimate the likelihood that the shared alleles are inherited from a common ancestor. The variable π, where $\pi =\frac{P\left(z=1\right)}{2}+P\left(z=2\right)$, represents the proportion of alleles shared IBD, and Z represents the IBD states of 0, 1, and 2 (Purcell et al., 2007). IBD was used for quality assessment of the dataset identifying any potential sample replicates or first-degree relatives. As the objective of this project analysis was to investigate overall population structure, this dataset was selected to avoid first- and second-degree relations that were supported by the IBD analysis. All pairwise comparisons within a breed were averaged to give an estimation of IBD within a breed.

Golden Helix SVS provides both a fixation index calculation which is equivalent to Wright’s F-statistic of F_ST_ as well as the co-ancestry coefficient θ, and an inbreeding estimation, *f*, which is equivalent to Wright’s F-statistic F_IS_ (Golden_Helix, 2012). For simplicity, the fixation index will be denoted as F_ST_ and the inbreeding coefficient will be denoted as *f* throughout the manuscript. The fixation index (F_ST_) is a measure of genetic divergence among subpopulation and ranges from 0 to 1 with 1 representing complete genetic divergence. The inbreeding coefficient, *f*, is a measure of an individual’s inbreeding with values ranging from −1 representing an excess of heterozygosity whereas +1 represents an excess of homozygosity. A value of 0 for *f* represents Hardy-Weinberg equilibrium. Individual inbreeding coefficients were determined for all animals with an average calculated for each breed.

In addition, an estimation of inbreeding was generated using the analysis results identifying individual ROH. Here, the equation,

$$F_{\text{ROH}}=\sum \frac{L_{\text{ROH}}}{L_{\text{AUTO}}}$$

from Purfield et al. (2012) was used where F_ROH_ is the estimate of inbreeding calculated using ROH, L_ROH_ is the sum of ROH per animal above the specified ROH length criteria, and L_AUTO_ is the total length of autosome covered by the SNPs. L_AUTO_ was 2,510,611 for the Illumina Bovine HD beadchip. The length criteria for L_ROH_ was >0.5 and >10 Mb for comparison to Purfield et al. (2012).

## Results

### JH1

While the majority of this study focused on the population structure of Island Jerseys, an important concern of all Jersey breeders is the recessive haplotype affecting fertility reported in 2011 (Van Raden et al., 2011), identified as Jersey Haplotype 1, or JH1. The putative causative nonsense mutation in *CWC15* was reported in 2013 by Sonstegard et al. (2013) and traced to a single ancestor, Observer Chocolate Soldier, a bull born in the United States in 1962 and registered with the American Jersey Cattle Association. The JH1 mutation was not found in any of the 39 genotyped Island Jersey bulls, supporting a premise that the mutation potentially developed in the United States population. Pedigree evaluation of the first three generations of Observer Chocolate Soldier identified the complete pedigree except for the paternal great grandsire. The animals that had unrecorded parents in the Observer pedigree were all born in the 1940s, with the most recent born in 1947. All animals in the pedigree were registered in the United States herdbook. There were no individuals with Island Jersey in the ancestry of Observer since the 1940s. With a relatively high carrier frequency in the United States Jersey population of 23.4% (Sonstegard et al., 2013), results from this study suggest that the popular Island Jersey bulls from 1964 to 2004 were not carriers of the JH1 mutation. Screening of the current Island Jersey population and imported germplasm to Jersey Island will allow breeders to maintain this status and provide a gene pool free of the JH1 mutation for international use.

### Y-Chromosome Lineages

For each animal Y-chromosome haplotype was determined using nine Y-specific markers. Two distinct haplotypes were found. One very common haplotype to the Jersey breed (Boichard et al., 2012), was most prevalent in Island Jersey samples. The second haplotype, however, was not present in Bovine HapMap animals and was unique to two Island Jersey individuals. The common Jersey haplotype differed by a single marker from the sole haplotype found in the single Guernsey bull as well as many other breeds from West Central Europe. Previous studies of Y-chromosome haplotypes in Jersey cattle support distinct patterns from those of Holstein (Edwards et al., 2011) as well as some similarities (Boichard et al., 2012). Six of the United States Jersey shared the nine Y-chromosome haplotype with Holstein as previously described. But, these haplotypes can be distinguished from Holstein by using additional Y-chromosome markers that are not available on the standard BovineHD manifest. The Island Jersey demonstrated no shared Y- chromosome haplotype with Holstein.

### Principal Component Analysis

**FIGURE 1:**
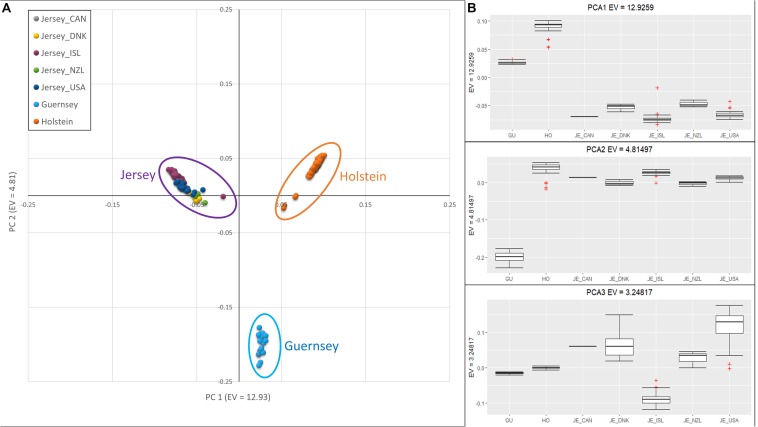
Principal Component Analysis showing clustering reflective of breed and sub-population. **(A)** Scatter plot of PCA with PC 1 on the *x*-axis, distinguishing Holstein (right-orange) and Jersey subpopulations (left-purple). PC 2 on the *y*-axis, distinguishes Guernsey from the other breeds. **(B)** Box-plots showing variation of principal components 1 (top), 2 (middle), and 3 (bottom) with individuals grouped by breed; Holstein and Guernsey or Jersey sub-populations; Danish (DNK), Island (ISL), New Zealand (NZL), and United States (USA). The ^+^ denote outlier samples in the dataset.

Jersey cattle originating from herds on Jersey Island, the United States, Canada, New Zealand, and Denmark were compared to the Holstein and Guernsey breeds using principal component analysis (PCA) and demonstrated breed homogeneity despite a range of geographic origins. The results from PCA are shown in Figure 1. The first principal component (PC1) had an eigenvalue of 12.93, and this component was associated with all three breeds, with the Holstein and Jersey breeds separated by the most extreme values and Guernsey breed intermediate. PC2, with an eigenvalue of 4.81, distinguished the Guernsey breed from the Jersey and Holstein breeds, and PC3 with an eigenvalue of 3.25, distinguished the Island Jersey from the rest of the cattle (Figure 1). The individual clustering of all three breeds support the notion of independent and unique breeds of cattle with generations of closed herdbooks and unique and breed-specific selection strategies. The geographic proximity of the two Channel Island breeds, Guernsey and Jersey, would suggest a closer genetic relationship between these breeds which was supported by the genetic principal component analyses (Figure 1B and Supplementary Figures S1, S2).

**FIGURE 2:**
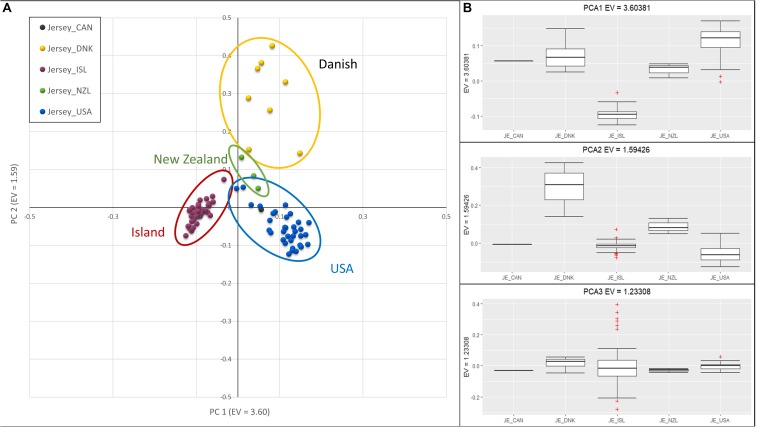
Principal Component Analysis of Jersey sub-populations based on country registration of animal. **(A)** Scatter plot with PC 1 on the *x*-axis, distinguishing Island (left-red) and United States (right-blue). PC 2 on the *y*-axis, distinguishes Danish. **(B)** Box-plots showing variation of principal components 1 (top), 2 (middle), and 3 (bottom) with individuals grouped by Jersey sub-populations; Canadian (CAN), Danish (DNK), Island (ISL), New Zealand (NZL), and United States (USA). The ^+^ denote outlier samples in the dataset.

Investigation of sub-structure within the Jersey breed using PCA produced clusters of individuals primarily reflecting country of origin, in particular, the grouping of Jersey Island animals away from the other geographic areas (Figure 2). However, sample size potentially biased clustering of Island and United States Jerseys in comparison to the extremely small sample sizes of animals from Canada, Denmark, and New Zealand. PC1, with an eigenvalue of 3.60, separated the Island from the remaining Jerseys. PC2 with an eigenvalue of 1.59, distinguished Jersey of Danish descent. Island Jerseys showed the highest degree of homogeneity as reflected in closer grouping of individual animals in the cluster. It is important to note that pedigree analysis was used to determine country of origin as opposed to country of registration. This approach was used to better attribute historic contributions from each country. Each of the breed societies represented here allow for offspring of a pure Jersey from one herdbook to be registered in another. As an example, one of the registered Canadian Jersey bull had all United States registered great-great grandparents (GGGP), yet four descendants of those 16 GGGP were registered as Canadian Jerseys, including two separate instances where two parents registered in the United States generated Canadian offspring. In addition, four cows registered in the United States were identified as Danish animals, because pedigree analysis of these individuals identified 8, 10, 11, and 13 GGGP of 16 possible animals identified as Danish. Interestingly, the animals registered in Denmark had 8, 8, and 12 Danish GGGP identified in their pedigrees. Animals with at least 4 GGGP from a country other than United States were designated as members of the foreign group of animals. As an example, one bull registered in the United States Jersey herdbook was designated as Danish in this study because he had 4 of 16 Danish GGGP. The loose clustering of the Danish animals likely reflects this rich interchange among countries of origin. The two Canadian registered Jerseys clustered with United States Jerseys and they had 0 and 4 of 16 GGGP identified as Canadian. The bull that had all 16 GGGP registered in the United States was considered as a United States S animal despite being registered in Canada. The three samples from New Zealand loosely clustered together and were intermediate to Island and United States Jerseys on PC1 and intermediate to the Danish and all other Jerseys on PC2. The three New Zealand Jersey had 10, 5, and 2 United States GGGP Jerseys which supports their intermediate clustering to the United States registered Jersey.

### Admixture Analysis

**FIGURE 3:**
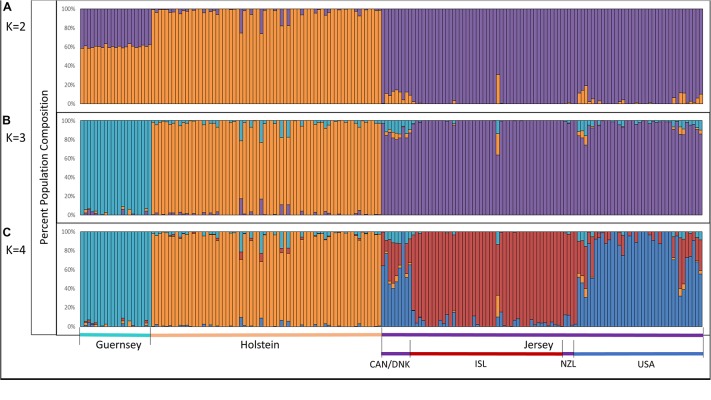
Admixture analysis reflecting genetic clustering of breed; Holstein, Guernsey, and Jersey and geographic origins of Jersey subpopulations; Island and United States. Individual vertical bars along the *x*-axis represent individual cattle which are grouped by breed. Genetic clusters corresponding to breed or population are denoted as follows: Guernsey (light/aqua blue), Holstein (orange), Jersey (purple), Island Jersey (ISL-red), and United States Jersey (USA-blue). Jersey cattle originating from Canada (CAN), Denmark (DNK), and New Zealand (NZL) are denoted in the bottom legend but do not show a unique genetic cluster. The *y*-axis provides a measure of the percentage of each genetic population found within an individual. *K* represents the number of genetic populations used in each analysis with **(A)** showing *K* = 2, **(B)** showing *K* = 3, and **(C)** showing *K* = 4.

**FIGURE 4:**
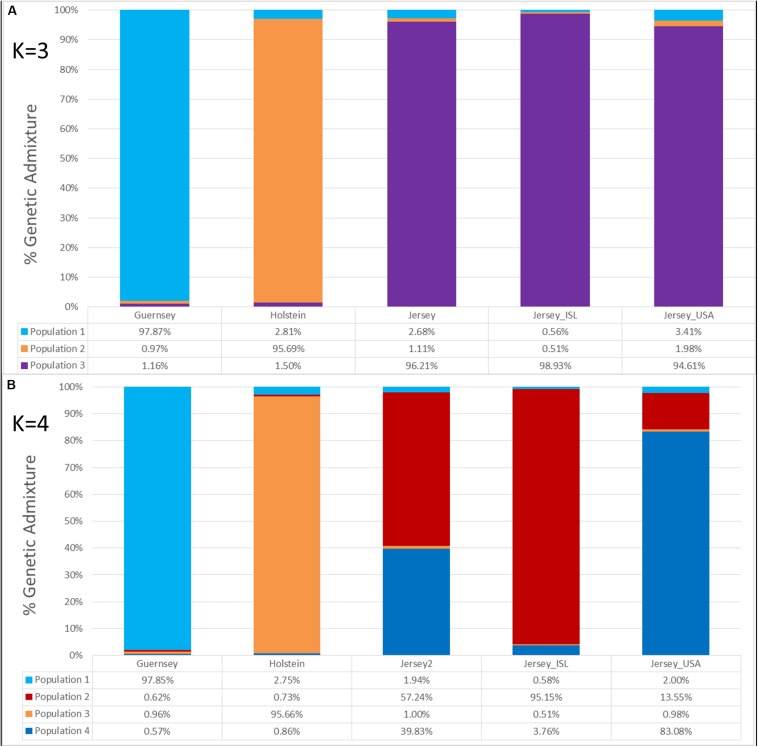
The average genetic admixture within each breed or Jersey subpopulation. Individual ADMIXTURE scores were averaged by breed or subpopulation designation. Genetic clusters corresponding to breed or population are denoted as follows: Guernsey (*n* = 21, light/aqua blue), Holstein (*n* = 65, orange), Jersey (*n* = 95, purple), Island Jersey (*n* = 49, ISL-red), and United States Jersey (*n* = 34, USA-blue). *K* represents the number of genetic populations used in each analysis with **(A)** showing *K* = 3 and **(B)** showing *K* = 4.

Admixture analysis was used to assess overall population structure comparing Holstein, Guernsey, and Jersey cattle as well as sub-structure and admixture within the Jersey breed. Cross-validation error calculations at *K* values of 2, 3, 4, and 5 genetic clusters predicted that a *K* of 4 showed the best predictive accuracy (*CV* = 0.53). Results describe the fraction or percentage of each unique genetic population identified in the admixture analysis for individual animals (Figure 3). The populations reflected the three breeds and Jersey subpopulations in the study as is typical for admixture analyses (Edea et al., 2013; Huson et al., 2014; O’Brien et al., 2015). For each individual, the fraction of that animal represented by each population was determined. Individual results were then classified by breed or subpopulation to generate average admixture estimates based on the genetic populations (Figure 4). The Holstein and Jersey breeds can be identified at a K of 2 with the Guernsey breed indicated at the K of 3 (Figures 3A,B). The distinction of the Holstein breed prior to the Guernsey breed in Figure 3 is likely a reflection of the Holstein breed (*n* = 65) having over three times the number of Guernsey breed (*n* = 21). ADMIXTURE analyses using equal representative numbers of individuals per breed (20 per breed) clearly segregated the Jersey at *K* = 2 but grouped the Holstein and Guernsey together (Supplementary Figure S3). Holstein and Guernsey cattle were slightly differentiated within this group as the Holstein had an average primary population value of 99% whereas the Guernsey had a slightly lower primary population value of 92%. The percentages reflect the similarity of individuals’ genotypes to the unique genetic patterns identified by ADMIXTURE software for the populations identified. The clear point exemplified at K of 3 is that individuals cluster respective to the reported breed that supports PCA results. Indeed, all but one individual had at least 96% genetic similarity to their designated breed. The single non-breed conforming individual was a United States Jersey and shows as an outlier in the PCA as well. Figures 3, 4 shows a slightly higher degree of admixture within individuals of the Holstein and Jersey breeds but again interpretation of this is likely over-represented due to an increased number of individuals representing these two breeds in comparison to the Guernsey breed. More importantly, a comparison of admixture within the Jersey breed shows greater homogeneity and less admixture within the Island Jersey (99% Jersey) as opposed to the United States Jersey (95% Jersey) (Figure 4A). The Island and United States Jersey segregate into different clusters at a *K* of 4, the optimal number of genetic clusters identified through cross-validation (*CV* = 0.53) (Figure 3C). Again, the Island Jersey show a higher degree of genetic homogeneity with an average of ∼95% of their genetic signature reflecting their Island origin and ∼4% reflecting a genetic similarity to the United States Jersey. In contrast, the United States Jersey average ∼83% of their genetic signature reflecting their United States origins with an additional ∼14% reflecting their similarity to the Island Jersey (Figure 4B). These results coincide with expectations of increased homogeneity within the Island Jersey due to their prolonged genetic isolation as well as a higher likelihood of potential admixture in the United States population. The fact that both Jersey subpopulations have some degree of the opposing Jersey subpopulation genetic signature supports breed uniformity of Jersey cattle. Likewise, the larger degree of Island Jersey genetic signature found in the United States Jersey supports a gene flow from the Island to the United States which corresponds to historical flow of germplasm.

### Signatures of Selection and Respective Gene Pathway Analysis

**FIGURE 5:**
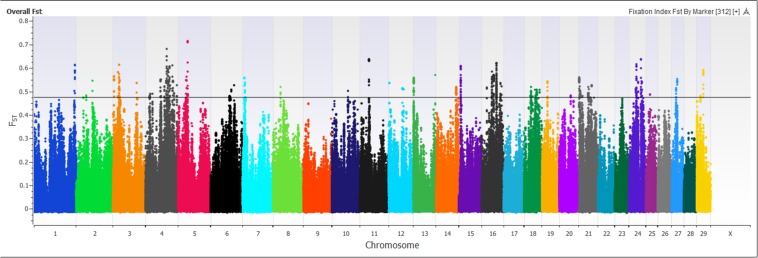
Signatures of selection are depicted using a Manhattan plot of marker-based F_ST_ values comparing Island Jersey and non-Island Jersey. The *x*-axis denotes the chromosome and the *y*-axis denotes the F_ST_ score for the SNP marker. The black horizontal line denotes five standard deviations above the mean marker F_ST_ value of 0.05.

A marker-based F_ST_ approach was used to identify regions of the genome showing genetic divergence, or selection, between the Island Jerseys and non-Island Jerseys (Figure 5). F_ST_ scores range from 0, representing no genetic divergence between sub-populations to 1, representing complete isolation or genetic divergence between sub-populations. 619,638 autosomal SNPs were evaluated in all Jersey cattle producing a mean marker F_ST_ of 0.06, with 29,716 markers showing no variation between populations while 307 markers had F_ST_ scores > 0.55 producing a standard deviation of 0.08. Significance of SNP F_ST_ was assessed by determining the mean and range of standard deviation. A single marker, BovineHD2400007509, had a F_ST_ score of 0.74, which was eight standard deviations above the mean. This marker was located on bovine chromosome 24 (BTA 24) at 27.5 Mb with multiple surrounding SNPs reaching the minimum 5 SD above the mean. The region contained three genes from the desmocollin gene family; *DSC1, DSC2*, and *DSC3*, all involved with epithelial proliferation, stratification, and differentiation (Legan et al., 1994). Thirty additional markers on BTA 3, 4, 5, 16, 21, and 24 were seven standard deviations above the mean (Supplementary Table S3). These SNPs were used to identify regions investigated in the PANTHER gene pathway analysis. Only 0.22% (*n* = 1,419) of the total SNPs were five standard deviations or above the mean F_ST_. PANTHER gene pathway analysis found no significant over- or under-representation of genes with associated biological processes in individual regions or a combined region analysis of F_ST_ locations. Thereby marker F_ST_ analysis identified multiple SNPs differentiating the Island and non-Island Jersey but biological significance of related areas was not identified.

### Runs of Homozygosity and Respective Gene Pathway Analysis

**FIGURE 6:**
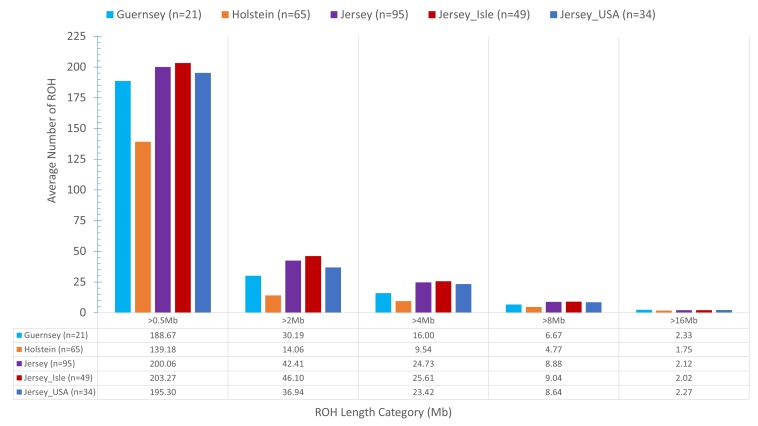
The mean sum of Run of Homozygosity (ROH) per genotyped animal within each population. The *x*-axis denotes the ROH length category (Mb) and the *y*-axis denotes the mean sum of ROH. Population designation is based on breed and Jersey sub-populations (ISL- Jersey Island, USA- United States) with unique color bars representing each population. Eighty-three individuals represented the Jersey breed. Actual mean sum of ROH are shown below the bar graph to assist in visualization of ROH lengths.

Identifying and comparing ROH within the Jersey breed, across sub-populations, and as compared to Holstein and Guernsey provided another method to identify genomic regions under potential selection or conservation as well as assessing inbreeding. On average, Jersey had a slightly higher number of short ROH (≤16 Mb) and an intermediate number of long range ROH (>16 Mb) than the average number of ROH per individual Holstein or Guernsey (Figure 6). In general, Holstein had fewer ROH of all lengths than Jersey or Guernsey, again demonstrating a higher degree of genetic similarity within the Channel Island breeds. Regions of the genome which commonly had ROH among the individuals analyzed were identified as a ROH cluster and analyzed for variation between the breeds and subpopulations and for biological significance. In total, 88 clusters of ROH were identified when assessing the 3 breeds together and 107 clusters were identified within the Jerseys with at least one cluster present on every autosomal chromosome in both analyses (Supplementary Tables S4, S5, respectively). The average length of a ROH cluster across the three breeds was 28 Mb (min = 1, max = 137 Mb) and contained an average of 6,997 SNPs (min = 40, max = 32,919 SNPs). Three ROH were found within at least 67% of all individuals representing the three breeds and identified on BTA 7:42.5–43.9, 16:42.8–43.6, and 16:43.8–45 Mb. PANTHER gene pathway analysis showed over-representation of genes associated with various lipid metabolic and cellular processes and immune function but neither the regional analysis nor a combined analysis provided results achieving significance after Bonferroni correction. The average length of a ROH cluster within Jerseys was slightly shorter at 23 Mb (min = 1, max = 131 Mb) and contained an average of 5,718 SNPs (min = 37, max = 31,483 SNPs). This is reflective of Jerseys having an increased number of short ROH. Ninety-three percent of the Jerseys carried the most common ROH identified within breed residing on BTA 21:31.9–32.4 Mb which was associated with fatty acid processes and generation of precursor metabolites and energy. None of these processes achieved significance after Bonferroni correction.

**FIGURE 7:**
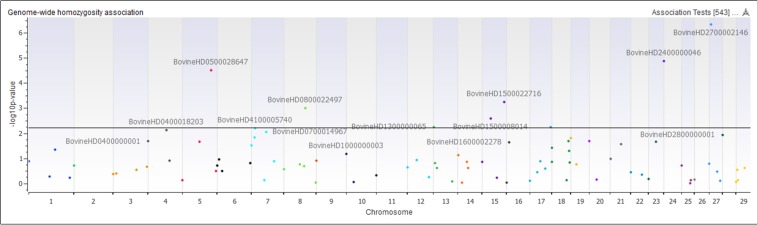
Genome-wide homozygosity association analysis comparing 107 runs of homozygosity clusters between Island and non-Island Jersey. Each dot represents the first SNP within the ROH cluster analyzed and is plotted according to genome location and level of association. Horizontal black line signifies a false discovery rate <0.05.

The genome-wide homozygosity association analysis comparing Island and non-Island Jerseys identified ten significantly associated ROH [false discovery rate (FDR) ≤ 0.5] (Figure 7 and Table 1). Eight of the ROH were present more frequently in the Island Jerseys, including the three most significantly associated regions on BTA 5, 24, and 27 (FDR ≤ 0.001). The Island Jerseys’ ROH on BTA 5:100,575,807–117,357,424 bp was significantly associated with complement activation (immune function) and blood coagulation and the ROH on BTA 7:51,524,490–79,119,815 bp was associated with anion transport. All other individual ROH cluster analyses showed associations with varying biological processes but did not achieve significance after Bonferroni correction. A combined analysis of all eight regions showed significant over-representation of genes in the biological processes of cell-cell signaling, sensory perception of chemical stimulus, and cell adhesion. Two regions had a higher prevalence in the non-Island Jerseys and were located on BTA 15 and 29. The fatty acid metabolic process was over-represented in genes on BTA 15 but did not achieve significance after Bonferroni correction. A combined analysis reduced this pathway association.

**TABLE 1:** Runs of homozygosity clusters identified as significantly associated with Jersey sub-populations.

		Position Start	End Position					
Chr	Start SNP	(bp)	(bp)	Length (bp)	# SNPs	*P*-value^1^	Population^2^	PANTHER Pathway^3^
5	BovineHD0500016748	59767596	99577125	39809529	9680	0.004010498	Island	Cholesterol metabolic processes
5	BovineHD0500028813	100575807	117357424	16781617	4362	3.02967E-05	Island	Complement activation*; Blood coagulation*
7	BovineHD0700014968	51524490	79119815	27595325	6783	0.000868087	Island	Anion transport*
8	BovineHD0800022594	75480001	110042671	34562670	7518	0.000855569	Island	Cell adhesion; Biological adhesion
13	BovineHD1300000156	921869	5332272	4410403	995	0.006151847	Island	Protein complex assembly
15	BovineHD1500008015	29876619	51078741	21202122	5538	0.002800973	Island	Blood circulation
15	BovineHD1500023615	81113269	85272311	4159042	1115	0.00151371	Non-Island	Fatty acid metabolic process
24	BovineHD2400000046	318334	62643699	62325365	15655	5.48781E-06	Island	Sensory perception of sound
27	BovineHD2700002147	6820125	28454147	21634022	5382	4.43187E-07	Island	Lipid metabolic process
29	BovineHD2900000137	988412	2010551	1022139	249	0.003766464	Non-Island	Amino acid transport

^1^Significance of association was validated with a false discovery rate of <0.05. ^2^The sub-population within which the ROH cluster occurred at greater frequency. ^3^The biological pathway identified by the PANTHER Classification System within each ROH. The biological processes within this column denoted by (*) reached a statistical significance of less than 0.05 after Bonferroni correction.

### Inbreeding Estimations

F-statistics and F_ROH_ estimations were calculated based on the study populations identified through breed and/or pedigree analysis. The Island and United States Jerseys were the only Jersey sub-populations analyzed for inbreeding as they were most comparable with similar numbers of animals and dates of birth (Supplementary Tables S1, S2). IBD calculations of pairs of individuals did not identify any sample duplication (pairwise IBD > 0.95). However, individuals with greater than 50% genetic similarity based on the markers analyzed were identified in each population suggesting first degree relationship between specific pairs of individuals. Guernsey had four individuals representing two pairwise comparisons with a maximum IBD score of 0.56. The Holstein breed had 39 individuals with a maximum pairwise IBD score of 0.53. Island and United States Jersey had 19 and 15 individuals, respectively with pairwise IBD scores greater than 0.5. Both populations had one pair of individuals with a maximum IBD score of 0.62. The average pairwise IBD score between individuals within each breed were 0.07 for Guernsey, 0.05 for Holstein, and 0.19 for Jersey.

**FIGURE 8:**
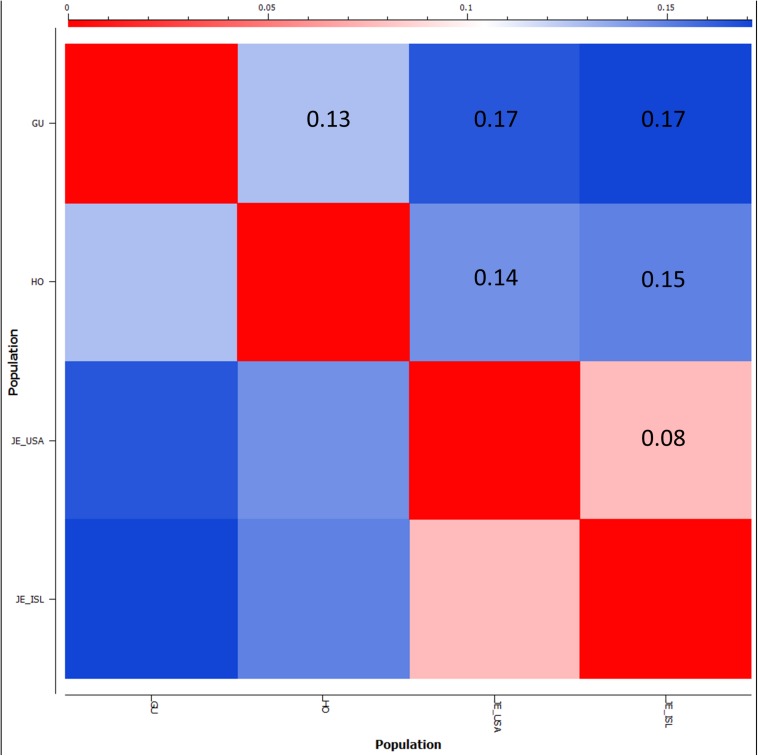
Population divergence between Guernsey, Holstein, and Jersey sub-populations based on fixation index F_ST_ scores. F_ST_ scores range from 0, no genetic divergence, to 1 representing complete genetic isolation. The matrix style heatmap shows pairwise comparisons of each population and colors range from red = 0 to blue = 0.17 (highest F_ST_ score).

F_ST_ was used to estimate genetic divergence between the Holstein, Guernsey, and the Jersey sub-populations (Figure 8). The Jersey sub-populations showed the lowest divergence amongst one another with a pairwise F_ST_ value of less than 0.08. This F_ST_ level is similar to that observed with other livestock breeds when comparing exported populations to the progenitor population (Blackburn et al., 2014). In contrast to expectation, Holsteins were more closely related to both the Guernseys (F_ST_ = 0.13) and Jerseys (F_ST_ ∼ 0.14) than the relationship between the two Channel Island breeds of Jersey and Guernsey (F_ST_ = 0.17). This is in contrast to typical research findings of genetic similarity reflecting geographic origins (Decker et al., 2014) and likely similar foundational stock during breed creation as supported by historical writings (RJA and HS, 2014; RGA and HS, 2016). While both PCA and F_ST_ provide measures of relationship, they are different. PCA determines the largest degree of variability within a dataset. Therefore, there is greater genetic variability between Holstein and Jersey cattle than between Guernsey and Jersey cattle. F_ST_ measures allele frequency divergence among sub-populations, thereby giving a measure of inbreeding among the sub-populations relative to the total population. F_ST_ statistics show less allele frequency divergence between the Channel Island breeds and Holsteins and greater divergence between the two Channel Island breeds themselves potentially reflecting admixture between the Holstein and international distribution of Guernsey and Jersey cattle. Overall, the difference in F_ST_ estimations between Jersey and Guernsey as opposed to Jersey and Holstein was only 0.03 with 95% confidence intervals ranging ±0.001. Future population analysis incorporating additional animals representing these breeds would provide validation of the current results and comparing these three breeds to additional breeds might clarify these results.

**FIGURE 9:**
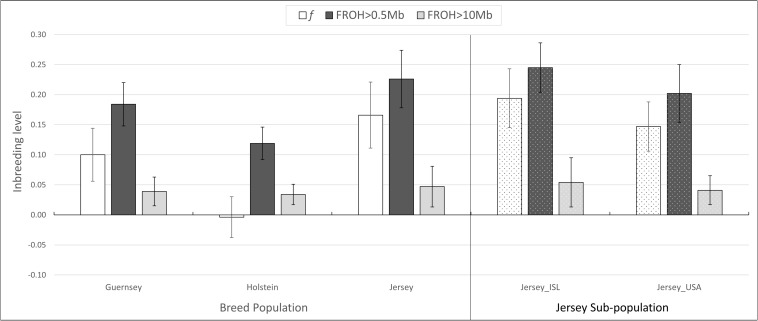
Average inbreeding estimations. Average inbreeding coefficient, *f*, and inbreeding estimate based on ROH measures, FROH. Population designation based on breed and pedigree for Jersey sub-populations. ISL, Jersey Island; USA, United States.

**FIGURE 10:**
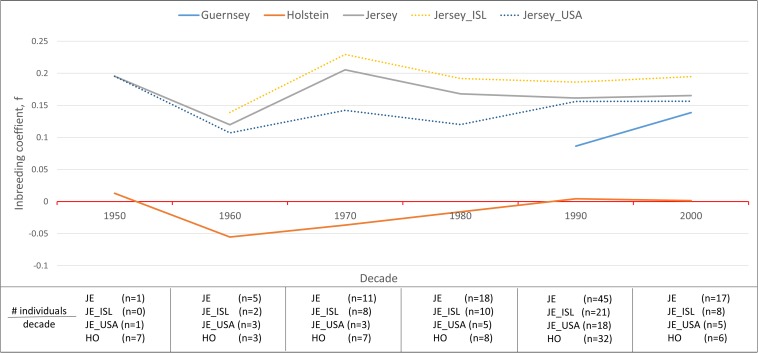
Variation in the average inbreeding coefficient, *f*, during each decade per population. Hardy-Weinberg Equilibrium is represented by a *f* value of zero (horizontal red line). Inbreeding coefficients below zero are representative of an excess of heterozygosity while those above zero are representative of an excess of homozygosity reflective of inbreeding. Breeds are represented by solid lines and Jersey sub-populations are represented by dotted lines. The absence of a line during a time period means that no individuals were analyzed from the said populations from that decade.

Inbreeding was calculated using the inbreeding coefficient (*f*) and estimated F_ROH_ (Figure 9). The average inbreeding coefficient per breed was lowest in Holstein (*f* = −0.004) and highest in Jersey (*f* = 0.166) cattle (Figure 9). These values are either similar or lower than other recent reports which is likely reflective of varying population sizes and origins and dependent upon the parameters used to calculate inbreeding (Bjelland et al., 2013). The overall conclusion of Holstein having lower average inbreeding as compared to Jersey coincides with recent reports (Stachowicz et al., 2011). To investigate the change in inbreeding over time, samples with birth date information were clustered by decade and used to generate an average *f* value per decade within each population (Figure 10). Only four of the 21 Guernsey cattle had known birthdates from the 1990’s and 2000’s. Two Holsteins had unknown birth dates. Overall, the Holstein and Jersey breeds and the Jersey subpopulations from the Island and United States had a similar number of bulls represented within each decade. All populations follow similar patterns of increasing and decreasing inbreeding over the decades (Figure 10). An evaluation of Island and United States Jerseys show similar average *f* scores of 0.194 and 0.147 yet achieving significant variation using a *T*-test comparison (*p*-value = 1.57 × 10^–5^).

Estimations of inbreeding using ROH also supported the above mentioned pattern with the Jersey breed having the highest inbreeding (F_ROH0.5_ = 0.22; F_ROH10_ = 0.5). Holstein again had the lowest inbreeding (F_ROH0.5_ = 0.12; F_ROH10_ = 0.03) (Figure 9). The variation in inbreeding levels found when using the 0.5 and 10 Mb ROH length thresholds for L_ROH_ show similar findings by Purfield et al. (2012). In general, the F_ROH_ estimations were generally higher than all other inbreeding measures when using the L_ROH_ of 0.5. Given that the Jersey had a higher number of short-range ROH, this likely influenced the higher F_ROH_ values using the 0.5 Mb threshold. Both Jersey subpopulations had an F_ROH_ greater than 0.2.

## Discussion

Over 200 years of breed development and exportation has seen the Jersey cattle adapt to varying regions with unique climates and production systems. Yet the founding population of Jersey cattle from the Island of Jersey has remained closed to genetic importation, therefore, maintaining strict breed purity and adapted solely to Island production. This study was designed to contrast the genetic difference between Island Jersey and non-Island Jersey cattle predominantly from the United States. It also compared basic population dynamics and inbreeding statistics of Jersey to Guernsey cattle originating on the neighboring Channel Island of Guernsey and the popular United States Holsteins. This research was largely driven by the 2008 legislation opening the Island of Jersey to germplasm importation in an effort to understand the foundation stock of Jersey cattle and future implications of gene flow. The foundation of the dataset analyzed consisted of 49 popular Jersey sires born between 1964 and 2004 from the Island of Jersey and comparable United States Jersey spanning the same decades. These data provided the first insights into the divergence of three major parts of the Jersey breed over decades of isolation; Island, United States, and Danish. Performance of these three subsets of the breed provide evidence of the measurable separation based on principal components in response to selection. The boundaries are sufficiently distinct that each of the three groups can be identified using non-overlapping borders. While the number of individuals used to characterize the populations were limited, these results provide compelling evidence for changes at the SNP level among the three largest groups.

Based on this study of popular Island Jersey sires, the JH1 mutation was not propagated by these bulls and was likely either eradicated due to its deleterious nature or not present in the Island population. The JHI recessive fertility haplotype is a particular health risk given the new gene flow into the Island Jersey population. Indeed, prior to JH1 being identified in 2011, two of the most popular international sires used upon early importation were JH1 carriers (Van Raden et al., 2011). The current frequency of the JH1 mutation in Island Jersey is unknown. The majority of Island dairy herd owners are actively selecting for JH1 free sires in their current breeding programs.

Principal component and admixture analysis of Jersey, Guernsey, and Holstein cattle demonstrated clustering of individuals reflective of breed designation (Figures 1, 3B). The Island Jerseys, most likely due to their genetic isolation, always segregated as the first sub-population within the Jersey breed (Figure 1). Marker-based F_ST_ values identified over 30 population informative SNPs for the Island Jersey (Figure 5 and Supplementary Table S3).

Another interesting finding of this research was the similar, yet statistically significant higher inbreeding estimates for the Island and United States Jersey populations likely reflecting drastically different population sizes (Figure 9). The United States has over 90 times the number of Jersey cattle as compared to the Island population and allows international gene flow within the breed. Our results support the expected higher degree of inbreeding in the Island Jersey due to the genetic isolation and relatively small total population. Inbreeding by decade shows a notable increase in inbreeding in the United States from 1980 to 1990 whereas inbreeding for the Island Jerseys showed a slight decline. Overall, Island Jerseys have maintained a consistent inbreeding level just below 0.2 since the 1980’s. New germplasm importation will likely decrease the Island Jerseys’ current inbreeding level and increase overall genetic diversity in the population.

An analysis of ROH and subsequent gene pathways provided insight into biological implications related to conserved regions found primarily in the Island Jerseys. In general, Jerseys have more ROH driven by a higher proportion of shorter length ROH (<8 Mb) commonly of ancestral origin (Figure 6; Keller et al., 2011; Kim et al., 2013). Common ROH among the three dairy breeds suggested increased selection for genes related to lipid metabolism whereas the most common ROH within the Jersey breed was over genes related to fatty-acid and energy metabolism. The two ROH clusters significantly associated with the non-Island Jerseys suggested biological pathways related to protein localization, cell cycle, and fatty acid metabolism (Table 1 and Figure 7). Eight ROH clusters were found significantly associated with the Island Jerseys and implicated biological pathways such as sensory perception, cell signaling and adhesion, anion transport, blood coagulation, and immune function after Bonferroni correction (Table 1 and Figure 7). Further research is needed to identify if this ancestral conservation of the Island Jersey genome is a product of adaptation to the Island production environment and to identify potential benefits. This project would benefit from validation of research results in the larger breeding cohort of Jersey cattle on the Island of Jersey and additional representatives of non-Island Jersey, Holstein and Guernsey cattle. This study highlights biological pathways and genes of significance in the Island Jerseys which may be affected by the influx of new genetics. Inbreeding measures, specific regions of the genome, and health or production traits related to the biological pathways identified can now be monitored in the Island population with earlier detection of both advantageous and deleterious changes. This in turn provides information for the future genetic management of the Island Jersey population.

## Data Availability Statement

The bovine genotypes representing the Guernsey, Holstein, and Jersey breeds are available through the Bovine Genome Database (https://bovinegenome.elsiklab.missouri.edu/) and the Council on Dairy Cattle Breeding Repository (https://www.uscdcb.com/). The bovine genotypes representing the Jersey Island cattle are available through the USDA National Animal Germplasm Program (https://agrin.ars.usda.gov/database_collaboration_page_dev).

## Author Contributions

Project conception and development was a collaborative effort among all authors. CV initiated the original project. HH compiled data, conducted all genetic analysis, and drafted the manuscript. TS processed JH1 results. JG, DH, and HB supplied much of the background information on the Island Jerseys whereas CW and GW added details for the United States Jerseys. All authors contributed to manuscript review.

## Conflict of Interest

TS was employed by USDA-ARS during the project initiation and analysis. TS was currently employed by the company Acceligen. The remaining authors declare that the research was conducted in the absence of any commercial or financial relationships that could be construed as a potential conflict of interest.
