# Supplementary material for: A Genetic Investigation of Island Jersey Cattle, the Foundation of the Jersey Breed: Comparing Population Structure and Selection to Guernsey, Holstein, and United States Jersey Cattle
Source: Front Genet. 2020 Apr 17;11:366. doi: 10.3389/fgene.2020.00366 (PMC7181675; doi:10.3389/fgene.2020.00366)
Supplement: Supplementary file 1 [file Data_Sheet_1.docx]

Supplementary Material

# Supplementary Tables

Supplementary Table 1. Cattle samples by breed per birth decade

| **Population** | **Decade of Birth** | | | | | | | |
| --- | --- | --- | --- | --- | --- | --- | --- | --- |
|  | 1950 | 1960 | 1970 | 1980 | 1990 | 2000 | Unknown | **Total** |
| **Guernsey** |  |  |  |  | 1 | 2 | 18 | **21** |
| **Holstein** | 7 | 3 | 7 | 8 | 32 | 6 | 2 | **65** |
| **Jersey** | 1 | 5 | 11 | 15 | 45 | 17 | 1 | **95** |
| ***Jersey_ISL*** |  | 2 | 8 | 10 | 21 | 8 | 0 | **49** |
| ***Jersey_USA*** | 1 | 3 | 3 | 5 | 18 | 4 | 0 | **34** |

Supplementary Table 2. Population sample sizes before and after quality assessment

| **Population^1^** | **Sample #** | **Sample # after QC^2^** |
| --- | --- | --- |
| Jersey_ISL | 49 | 49 |
| Jersey_USA | 39 | 34 |
| Jersey_CAN | 2 | 1 |
| Jersey_DNK | 3 | 8 |
| Jersey_NZL | 3 | 3 |
| Holstein | 71 | 65 |
| Guernsey | 21 | 21 |
| **Total** | **188** | **181** |

Population designation based on pedigree and registry assignments. ISL- Jersey Island, USA- United States, CAN- Canada, DNK- Danish, NZL- New Zealand

QC- Quality Control measures including call rate < 0.95 and Identity by Descent calculations. Dataset used for principal component analysis.

Supplementary Table 3. F_ST_ Identified Population Informative Markers

| **Marker** | **Chromosome** | **Position** | **Overall F_ST_** |
| --- | --- | --- | --- |
| BovineHD2400007509 | 24 | 27545449 | 0.740382 |
| BovineHD0500010049 | 5 | 34956360 | 0.699084 |
| BovineHD0500010045 | 5 | 34945553 | 0.694704 |
| BovineHD0500010052 | 5 | 34965958 | 0.694704 |
| BovineHD1600019489 | 16 | 68864523 | 0.690657 |
| BovineHD0500010053 | 5 | 34967024 | 0.686965 |
| BovineHD0500010054 | 5 | 34968278 | 0.686965 |
| BovineHD0500010056 | 5 | 34976064 | 0.686965 |
| BovineHD1600019488 | 16 | 68863373 | 0.684897 |
| BovineHD0300007453 | 3 | 23769237 | 0.683891 |
| BovineHD0500010051 | 5 | 34964194 | 0.683846 |
| BovineHD0500010050 | 5 | 34959585 | 0.679699 |
| BovineHD0500010055 | 5 | 34971353 | 0.675031 |
| BovineHD2100013876 | 21 | 48485558 | 0.672646 |
| BovineHD1600019700 | 16 | 69395350 | 0.666802 |
| BovineHD2400007420 | 24 | 27127417 | 0.662997 |
| BovineHD2400012516 | 24 | 45631017 | 0.661393 |
| BovineHD2100013877 | 21 | 48487618 | 0.660491 |
| BovineHD0400021862 | 4 | 78833183 | 0.659831 |
| BovineHD4100001926 | 3 | 22065634 | 0.659648 |
| BovineHD1600019815 | 16 | 69744456 | 0.659327 |
| BovineHD2400012520 | 24 | 45643939 | 0.657706 |
| BovineHD2400012521 | 24 | 45645044 | 0.656989 |
| BovineHD0300006927 | 3 | 22064019 | 0.656453 |
| BovineHD0300007450 | 3 | 23757088 | 0.654844 |
| BovineHD0500009190 | 5 | 31590800 | 0.649806 |
| BovineHD2400012519 | 24 | 45642076 | 0.647532 |
| BovineHD2400012522 | 24 | 45646650 | 0.647532 |
| ARS-BFGL-NGS-75337 | 24 | 28007169 | 0.647224 |
| BovineHD0400021970 | 4 | 79379288 | 0.645722 |
| BovineHD0300007452 | 3 | 23766067 | 0.642581 |

# Supplementary Table 4. 88 ROH Clusters Common Across Holstein, Guernsey, and Jersey.

| Cluster ID | Chr | Start SNP Name | Start Position (bp) | End Position (bp) | Length (bp) | # SNPs |
| --- | --- | --- | --- | --- | --- | --- |
| 1 | 1 | BovineHD0100000005 | 16947 | 74917513 | 74900566 | 18270 |
| 2 | 1 | BovineHD0100021507 | 75049489 | 93716184 | 18666695 | 3881 |
| 3 | 1 | BovineHD0100026852 | 94569153 | 145292228 | 50723075 | 12623 |
| 4 | 1 | BovineHD0100042170 | 146004225 | 158322647 | 12318422 | 3723 |
| 5 | 2 | ARS-BFGL-NGS-102158 | 35126 | 136776410 | 136741284 | 32919 |
| 6 | 2 | ARS-BFGL-NGS-22911 | 137692158 | 139987690 | 2295532 | 52 |
| 7 | 3 | BovineHD0300000003 | 25683 | 11983466 | 11957783 | 3438 |
| 8 | 3 | BovineHD4100001844 | 12329835 | 82701810 | 70371975 | 16570 |
| 9 | 3 | BovineHD0300023762 | 83029339 | 119462443 | 36433104 | 9706 |
| 10 | 3 | BovineHD0300034879 | 119490208 | 121408443 | 1918235 | 422 |
| 11 | 4 | BovineHD0400000001 | 1062 | 65642372 | 65641310 | 15040 |
| 12 | 4 | BovineHD0400018203 | 66279716 | 76746778 | 10467062 | 2500 |
| 13 | 4 | BovineHD0400021344 | 77112251 | 120625322 | 43513071 | 11927 |
| 14 | 5 | BovineHD0500000007 | 31485 | 58961352 | 58929867 | 13729 |
| 15 | 5 | BovineHD0500016665 | 59421039 | 99577125 | 40156086 | 9743 |
| 16 | 5 | BovineHD0500028647 | 100094027 | 117357424 | 17263397 | 4519 |
| 17 | 5 | BovineHD0500034197 | 117798518 | 121183174 | 3384656 | 1061 |
| 18 | 6 | BovineHD0600000005 | 31280 | 5298300 | 5267020 | 1239 |
| 19 | 6 | BovineHD0600001488 | 6870970 | 17915133 | 11044163 | 2992 |
| 20 | 6 | BovineHD0600004921 | 18038454 | 117045854 | 99007400 | 25400 |
| 21 | 6 | BovineHD0600033303 | 117060333 | 119454666 | 2394333 | 771 |
| 22 | 7 | BovineHD0700000001 | 10880 | 10722414 | 10711534 | 2111 |
| 23 | 7 | BovineHD0700002840 | 10867506 | 11555132 | 687626 | 58 |
| 24 | 7 | BovineHD4100005740 | 12217156 | 44176069 | 31958913 | 8107 |
| 25 | 7 | BovineHD0700012841 | 44542592 | 51279740 | 6737148 | 1573 |
| 26 | 7 | BovineHD0700014967 | 51520623 | 79119815 | 27599192 | 6784 |
| 27 | 7 | BovineHD0700023224 | 79550184 | 112628884 | 33078700 | 8421 |
| 28 | 8 | BovineHD0800000001 | 20855 | 56712926 | 56692071 | 12415 |
| 29 | 8 | BovineHD0800017108 | 56960163 | 70798343 | 13838180 | 2820 |
| 30 | 8 | BovineHD0800021427 | 71267977 | 74479423 | 3211446 | 641 |
| 31 | 8 | BovineHD0800022497 | 75176717 | 110042671 | 34865954 | 7594 |
| 32 | 8 | BTB-00376866 | 111945098 | 113190754 | 1245656 | 40 |
| 33 | 9 | BovineHD0900031325 | 10049 | 105688974 | 105678925 | 26037 |
| 34 | 10 | BovineHD1000000003 | 23914 | 22512596 | 22488682 | 6590 |
| 35 | 10 | BovineHD1000008266 | 25327628 | 104301732 | 78974104 | 19736 |
| 36 | 11 | BovineHD1100000002 | 20031 | 107282960 | 107262929 | 28076 |
| 37 | 12 | BovineHD1200000001 | 6351 | 32020724 | 32014373 | 8399 |
| 38 | 12 | BovineHD1200009830 | 33508422 | 70376431 | 36868009 | 8554 |
| 39 | 12 | BovineHD1200021802 | 77177343 | 91105777 | 13928434 | 4375 |
| 40 | 13 | BovineHD1300000065 | 416621 | 5332272 | 4915651 | 1061 |
| 41 | 13 | BovineHD1300001411 | 5628236 | 10966294 | 5338058 | 1284 |
| 42 | 13 | BovineHD1300003132 | 11516469 | 64660314 | 53143845 | 11042 |
| 43 | 13 | Hapmap48297-BTA-33286 | 64977414 | 84229982 | 19252568 | 4054 |
| 44 | 14 | BovineHD1400000076 | 1118964 | 14482826 | 13363862 | 3599 |
| 45 | 14 | BovineHD1400004412 | 15726111 | 32304518 | 16578407 | 3671 |
| 46 | 14 | BTB-01689254 | 32569930 | 36995098 | 4425168 | 834 |
| 47 | 14 | Hapmap41415-BTA-107703 | 37535038 | 84628243 | 47093205 | 10013 |
| 48 | 15 | BovineHD1500000003 | 43737 | 29532474 | 29488737 | 6923 |
| 49 | 15 | BovineHD1500008014 | 29871540 | 51078741 | 21207201 | 5539 |
| 50 | 15 | BovineHD1500014795 | 51502997 | 77788979 | 26285982 | 7037 |
| 51 | 15 | BovineHD1500022716 | 77993255 | 85272311 | 7279056 | 1746 |
| 52 | 16 | BovineHD1600000002 | 35920 | 6897746 | 6861826 | 1515 |
| 53 | 16 | BovineHD1600002278 | 7949464 | 81672961 | 73723497 | 18896 |
| 54 | 17 | BovineHD1700000002 | 2892 | 25010293 | 25007401 | 6393 |
| 55 | 17 | BovineHD1700007081 | 25212697 | 35637592 | 10424895 | 2441 |
| 56 | 17 | BovineHD1700009964 | 36596129 | 50696256 | 14100127 | 3971 |
| 57 | 17 | BovineHD1700014659 | 52369600 | 72808655 | 20439055 | 5806 |
| 58 | 17 | BovineHD1700021320 | 72949950 | 75132928 | 2182978 | 437 |
| 59 | 18 | BovineHD1800000009 | 85883 | 657343 | 571460 | 49 |
| 60 | 18 | BovineHD1800000093 | 835815 | 50870670 | 50034855 | 13863 |
| 61 | 18 | BovineHD1800015044 | 51373488 | 57385696 | 6012208 | 1466 |
| 62 | 18 | BovineHD1800016890 | 58004859 | 61722654 | 3717795 | 553 |
| 63 | 18 | BovineHD1800017874 | 61988764 | 63172836 | 1184072 | 387 |
| 64 | 18 | BovineHD1800018375 | 63452775 | 65999195 | 2546420 | 726 |
| 65 | 19 | BovineHD1900000003 | 90671 | 19795016 | 19704345 | 5182 |
| 66 | 19 | BovineHD1900005719 | 20001162 | 64044783 | 44043621 | 11608 |
| 67 | 20 | BovineHD2000000006 | 49079 | 25072887 | 25023808 | 6775 |
| 68 | 20 | BovineHD2000007590 | 25323795 | 71986227 | 46662432 | 12098 |
| 69 | 21 | BovineHD2100000257 | 2082072 | 37734859 | 35652787 | 9181 |
| 70 | 21 | BovineHD2100011048 | 37953253 | 71573501 | 33620248 | 8633 |
| 71 | 22 | BovineHD2200000013 | 92533 | 39725145 | 39632612 | 10294 |
| 72 | 22 | BovineHD4100015677 | 40150026 | 61379134 | 21229108 | 5960 |
| 73 | 23 | BovineHD2300000001 | 10121 | 25669376 | 25659255 | 6557 |
| 74 | 23 | BovineHD2300007255 | 26784050 | 52465632 | 25681582 | 6817 |
| 75 | 24 | BovineHD2400000046 | 318334 | 62643699 | 62325365 | 15655 |
| 76 | 25 | BovineHD2500000005 | 25945 | 30269233 | 30243288 | 8237 |
| 77 | 25 | BovineHD2500008467 | 30526710 | 32360791 | 1834081 | 487 |
| 78 | 25 | BovineHD2500008985 | 32763101 | 42851121 | 10088020 | 2778 |
| 79 | 26 | BovineHD2600000073 | 1016728 | 51674807 | 50658079 | 13604 |
| 80 | 27 | BovineHD2700000001 | 2673 | 5128900 | 5126227 | 1485 |
| 81 | 27 | BovineHD2700002146 | 6817065 | 28454147 | 21637082 | 5383 |
| 82 | 27 | BovineHD2700007982 | 28941472 | 37557668 | 8616196 | 2540 |
| 83 | 27 | BovineHD2700011038 | 38278231 | 45402893 | 7124662 | 1968 |
| 84 | 28 | BovineHD2800000001 | 5302 | 46248750 | 46243448 | 11719 |
| 85 | 29 | BovineHD2900000137 | 988412 | 5440022 | 4451610 | 1236 |
| 86 | 29 | BovineHD2900001653 | 5884042 | 6580683 | 696641 | 137 |
| 87 | 29 | BovineHD2900001832 | 6622122 | 30221261 | 23599139 | 6505 |
| 88 | 29 | BovineHD2900009275 | 31148059 | 51499351 | 20351292 | 4693 |

# Supplementary Table 5. 107 ROH Clusters Common Across Jersey Subpopulations.

| Cluster ID | Chr | Start SNP Name | Start Position (bp) | End Position (bp) | Length (bp) | # SNPs |
| --- | --- | --- | --- | --- | --- | --- |
| 1 | 1 | BovineHD0100000005 | 16947 | 74917513 | 74900566 | 18270 |
| 2 | 1 | BovineHD0100021507 | 75049489 | 93716184 | 18666695 | 3881 |
| 3 | 1 | BovineHD0100026855 | 94581777 | 145292228 | 50710451 | 12620 |
| 4 | 1 | BovineHD0100042195 | 146114076 | 158322647 | 12208571 | 3708 |
| 5 | 2 | BovineHD0200000023 | 174818 | 2216441 | 2041623 | 553 |
| 6 | 2 | BovineHD0200000633 | 2274183 | 3668510 | 1394327 | 275 |
| 7 | 2 | BovineHD0200001097 | 4087924 | 135202117 | 131114193 | 31483 |
| 8 | 2 | BovineHD0200039555 | 135210052 | 135720704 | 510652 | 178 |
| 9 | 2 | BovineHD0200039786 | 135813058 | 136776410 | 963352 | 241 |
| 10 | 2 | ARS-BFGL-NGS-22911 | 137692158 | 139987690 | 2295532 | 52 |
| 11 | 3 | BovineHD0300000003 | 25683 | 11983466 | 11957783 | 3438 |
| 12 | 3 | BovineHD4100001844 | 12329835 | 54414408 | 42084573 | 10586 |
| 13 | 3 | BovineHD0300016527 | 54593967 | 82701810 | 28107843 | 5974 |
| 14 | 3 | Hapmap40290-BTA-68546 | 83574052 | 119461017 | 35886965 | 9570 |
| 15 | 3 | BovineHD0300035001 | 119762038 | 121408443 | 1646405 | 321 |
| 16 | 4 | BovineHD0400000001 | 1062 | 65642372 | 65641310 | 15040 |
| 17 | 4 | BovineHD4100002964 | 66509280 | 76746778 | 10237498 | 2452 |
| 18 | 4 | BovineHD0400021354 | 77123813 | 120625322 | 43501509 | 11918 |
| 19 | 5 | BovineHD0500000007 | 31485 | 1071201 | 1039716 | 173 |
| 20 | 5 | BovineHD0500000248 | 1082277 | 58961352 | 57879075 | 13555 |
| 21 | 5 | BovineHD0500016748 | 59767596 | 99577125 | 39809529 | 9680 |
| 22 | 5 | BovineHD0500028813 | 100575807 | 117357424 | 16781617 | 4362 |
| 23 | 5 | BovineHD0500034335 | 118219758 | 121183174 | 2963416 | 935 |
| 24 | 6 | BovineHD0600000005 | 31280 | 5298300 | 5267020 | 1239 |
| 25 | 6 | BovineHD0600001553 | 7153235 | 7852787 | 699552 | 206 |
| 26 | 6 | BovineHD0600001780 | 7857073 | 17915133 | 10058060 | 2753 |
| 27 | 6 | BovineHD0600004921 | 18038454 | 106958735 | 88920281 | 22645 |
| 28 | 6 | BovineHD0600030164 | 107210049 | 117045854 | 9835805 | 2679 |
| 29 | 6 | BovineHD0600033326 | 117127260 | 119454666 | 2327406 | 751 |
| 30 | 7 | BovineHD0700000001 | 10880 | 6718398 | 6707518 | 1656 |
| 31 | 7 | BovineHD0700002047 | 8058835 | 9776185 | 1717350 | 349 |
| 32 | 7 | BovineHD0700002614 | 9968052 | 10722414 | 754362 | 49 |
| 33 | 7 | BovineHD0700002840 | 10867506 | 11555132 | 687626 | 58 |
| 34 | 7 | BovineHD0700003249 | 12506524 | 13145679 | 639155 | 91 |
| 35 | 7 | BovineHD0700003520 | 13434786 | 44176069 | 30741283 | 7861 |
| 36 | 7 | BovineHD0700012859 | 44585217 | 51279740 | 6694523 | 1559 |
| 37 | 7 | BovineHD0700014968 | 51524490 | 79119815 | 27595325 | 6783 |
| 38 | 7 | BovineHD0700023224 | 79550184 | 112628884 | 33078700 | 8421 |
| 39 | 8 | BovineHD0800000001 | 20855 | 35671830 | 35650975 | 7828 |
| 40 | 8 | BovineHD0800010673 | 35947535 | 56712926 | 20765391 | 4529 |
| 41 | 8 | BovineHD0800017108 | 56960163 | 70798343 | 13838180 | 2820 |
| 42 | 8 | BovineHD0800021452 | 71303084 | 74479423 | 3176339 | 617 |
| 43 | 8 | BovineHD0800022594 | 75480001 | 110042671 | 34562670 | 7518 |
| 44 | 8 | BTB-00376866 | 111945098 | 113118348 | 1173250 | 37 |
| 45 | 9 | BovineHD0900031325 | 10049 | 105688974 | 105678925 | 26037 |
| 46 | 10 | BovineHD1000000003 | 23914 | 22512596 | 22488682 | 6590 |
| 47 | 10 | BovineHD1000008482 | 26097706 | 104301732 | 78204026 | 19570 |
| 48 | 11 | BovineHD1100000002 | 20031 | 76741330 | 76721299 | 19788 |
| 49 | 11 | BovineHD1100021974 | 76791067 | 107282960 | 30491893 | 8277 |
| 50 | 12 | BovineHD1200000001 | 6351 | 32020724 | 32014373 | 8399 |
| 51 | 12 | BovineHD1200010153 | 34564169 | 35094683 | 530514 | 141 |
| 52 | 12 | BovineHD1200010404 | 35477977 | 70376431 | 34898454 | 8056 |
| 53 | 12 | BovineHD1200021804 | 77186525 | 91105777 | 13919252 | 4373 |
| 54 | 13 | BovineHD1300000156 | 921869 | 5332272 | 4410403 | 995 |
| 55 | 13 | BovineHD1300001441 | 5718711 | 10966294 | 5247583 | 1252 |
| 56 | 13 | BovineHD1300003132 | 11516469 | 64660314 | 53143845 | 11042 |
| 57 | 13 | Hapmap48297-BTA-33286 | 64977414 | 84229982 | 19252568 | 4054 |
| 58 | 14 | BovineHD1400024034 | 1260498 | 14482826 | 13222328 | 3597 |
| 59 | 14 | BovineHD1400004427 | 15783379 | 32304518 | 16521139 | 3659 |
| 60 | 14 | BTB-01689254 | 32569930 | 36995098 | 4425168 | 834 |
| 61 | 14 | Hapmap41415-BTA-107703 | 37535038 | 84628243 | 47093205 | 10013 |
| 62 | 15 | BovineHD1500000003 | 43737 | 29532474 | 29488737 | 6923 |
| 63 | 15 | BovineHD1500008015 | 29876619 | 51078741 | 21202122 | 5538 |
| 64 | 15 | BovineHD1500025869 | 51653453 | 77788979 | 26135526 | 6981 |
| 65 | 15 | BovineHD1500022716 | 77993255 | 81042220 | 3048965 | 608 |
| 66 | 15 | BovineHD1500023615 | 81113269 | 85272311 | 4159042 | 1115 |
| 67 | 16 | BovineHD1600000002 | 35920 | 6897746 | 6861826 | 1515 |
| 68 | 16 | BovineHD1600002278 | 7949464 | 45392567 | 37443103 | 9016 |
| 69 | 16 | BovineHD1600012619 | 45655772 | 81672961 | 36017189 | 9879 |
| 70 | 17 | BovineHD1700000002 | 2892 | 25010293 | 25007401 | 6393 |
| 71 | 17 | BovineHD1700007121 | 25330031 | 35637592 | 10307561 | 2402 |
| 72 | 17 | BovineHD1700010031 | 36824235 | 50696256 | 13872021 | 3909 |
| 73 | 17 | BovineHD1700014684 | 52415901 | 72808655 | 20392754 | 5787 |
| 74 | 17 | BovineHD1700021320 | 72949950 | 75132928 | 2182978 | 437 |
| 75 | 18 | BovineHD1800000009 | 85883 | 657343 | 571460 | 49 |
| 76 | 18 | BovineHD1800000614 | 2255379 | 50870670 | 48615291 | 13416 |
| 77 | 18 | BovineHD1800015044 | 51373488 | 57385696 | 6012208 | 1466 |
| 78 | 18 | BovineHD1800016890 | 58004859 | 61722654 | 3717795 | 553 |
| 79 | 18 | BovineHD1800017956 | 62242886 | 63172836 | 929950 | 321 |
| 80 | 18 | BovineHD1800018433 | 63644604 | 65999195 | 2354591 | 689 |
| 81 | 19 | BovineHD1900000003 | 90671 | 19795016 | 19704345 | 5182 |
| 82 | 19 | BovineHD1900005719 | 20001162 | 64044783 | 44043621 | 11608 |
| 83 | 20 | BovineHD2000000006 | 49079 | 25072887 | 25023808 | 6775 |
| 84 | 20 | BovineHD2000007590 | 25323795 | 71986227 | 46662432 | 12098 |
| 85 | 21 | BovineHD2100000257 | 2082072 | 2653550 | 571478 | 64 |
| 86 | 21 | BovineHD2100000368 | 2734021 | 37734859 | 35000838 | 9109 |
| 87 | 21 | BovineHD2100011082 | 38084069 | 71573501 | 33489432 | 8599 |
| 88 | 22 | BovineHD2200000013 | 92533 | 39725145 | 39632612 | 10294 |
| 89 | 22 | BovineHD2200011437 | 40172586 | 61379134 | 21206548 | 5951 |
| 90 | 23 | BovineHD2300000001 | 10121 | 25669376 | 25659255 | 6557 |
| 91 | 23 | BovineHD2300007319 | 26929522 | 52465632 | 25536110 | 6770 |
| 92 | 24 | BovineHD2400000046 | 318334 | 62643699 | 62325365 | 15655 |
| 93 | 25 | BovineHD2500000005 | 25945 | 30269233 | 30243288 | 8237 |
| 94 | 25 | BovineHD2500008467 | 30526710 | 32360791 | 1834081 | 487 |
| 95 | 25 | BovineHD2500008986 | 32768835 | 42851121 | 10082286 | 2777 |
| 96 | 26 | BovineHD2600000073 | 1016728 | 25790658 | 24773930 | 5983 |
| 97 | 26 | BovineHD2600006849 | 25997857 | 51674807 | 25676950 | 7567 |
| 98 | 27 | BovineHD2700000001 | 2673 | 5128900 | 5126227 | 1485 |
| 99 | 27 | BovineHD2700002147 | 6820125 | 28454147 | 21634022 | 5382 |
| 100 | 27 | BovineHD2700007987 | 28954158 | 35555613 | 6601455 | 1918 |
| 101 | 27 | BovineHD2700011055 | 38323585 | 45402893 | 7079308 | 1951 |
| 102 | 28 | BovineHD2800000001 | 5302 | 46248750 | 46243448 | 11719 |
| 103 | 29 | BovineHD2900000137 | 988412 | 2010551 | 1022139 | 249 |
| 104 | 29 | BovineHD4100018792 | 2196297 | 5440022 | 3243725 | 940 |
| 105 | 29 | BovineHD2900001893 | 6782632 | 30221261 | 23438629 | 6446 |
| 106 | 29 | BovineHD2900009293 | 31204866 | 33170858 | 1965992 | 538 |
| 107 | 29 | BovineHD2900009877 | 33183593 | 51499351 | 18315758 | 4134 |

# Supplementary Figures


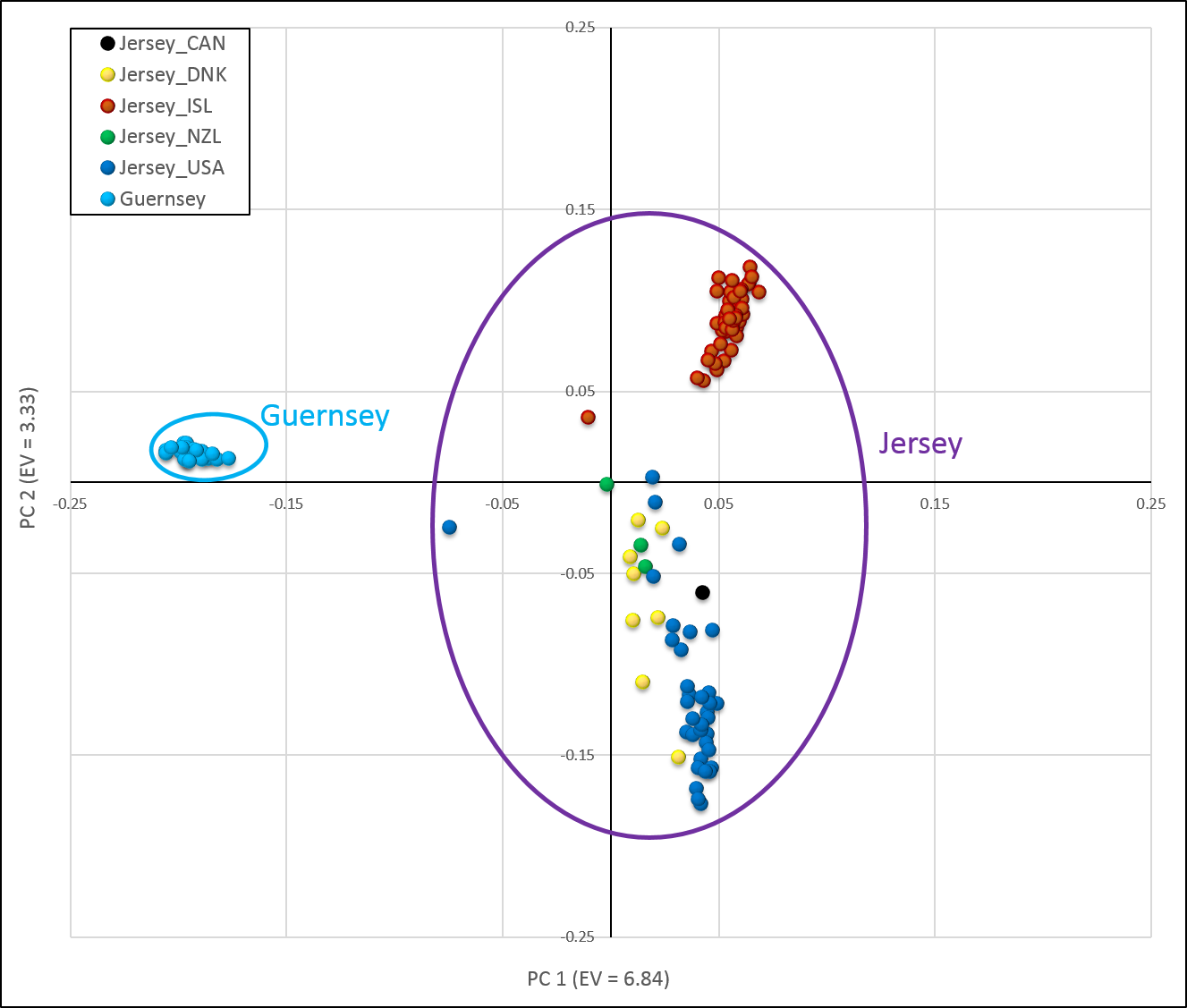


**Supplementary Figure 1.** Principle Component Analysis of Jersey sub-populations based on country registration of animal and Guernsey. PC 1, x-axis, distinguishes the Guernsey (left- light blue) from the Jersey subpopulations (right). PC 2, y-axis, distinguishes the Island Jersey (top right- red) primarily from the US Jersey (bottom right- dark blue).


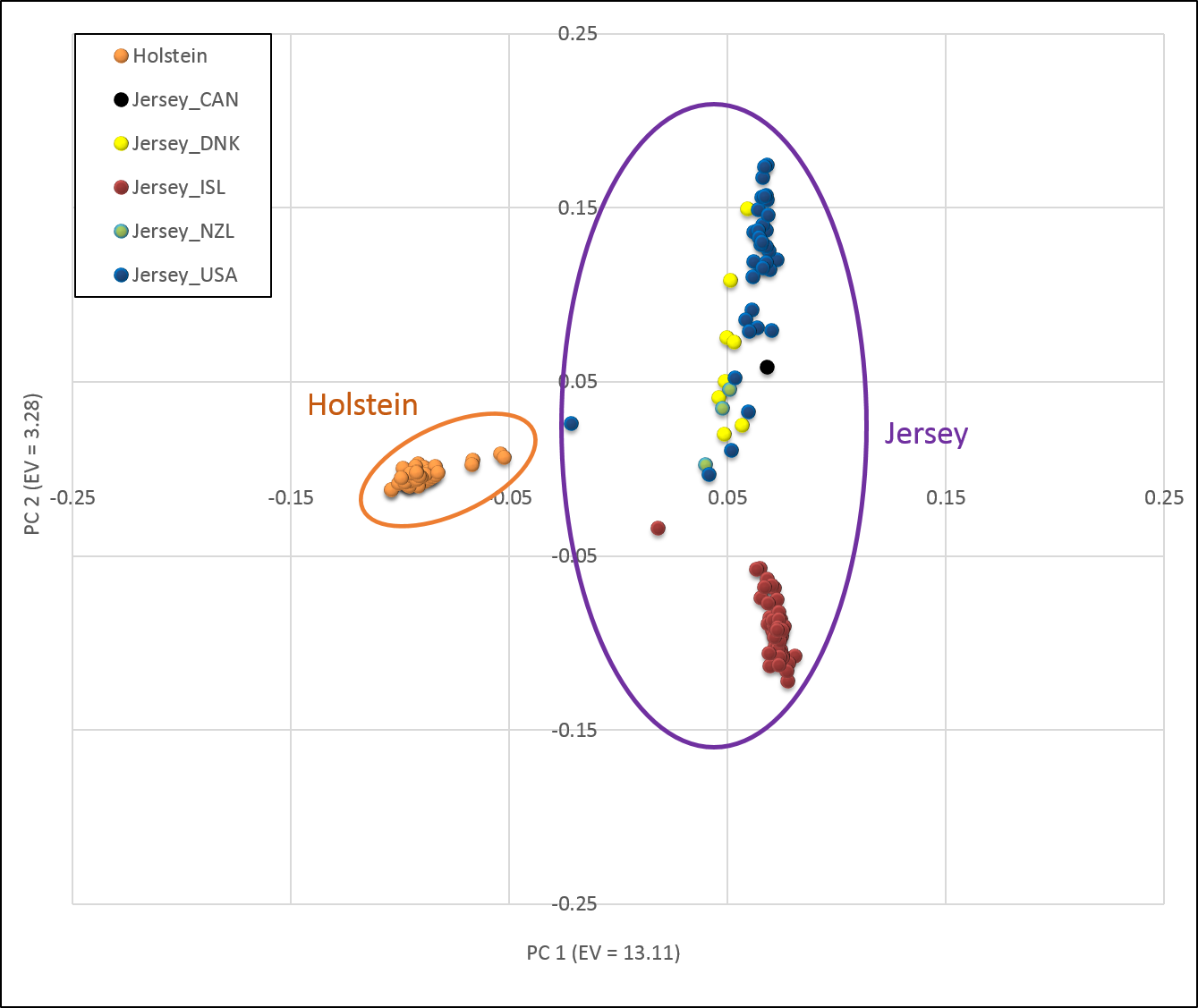


**Supplemental Figure 2.** Principle Component Analysis of Jersey sub-populations based on country registration of animal and Holstein. PC 1, x-axis, distinguishes the Holstein (left- orange) from the Jersey subpopulations (right). PC 2, y-axis, distinguishes the Island Jersey (bottom right- red) primarily from the US Jersey (top right- dark blue).


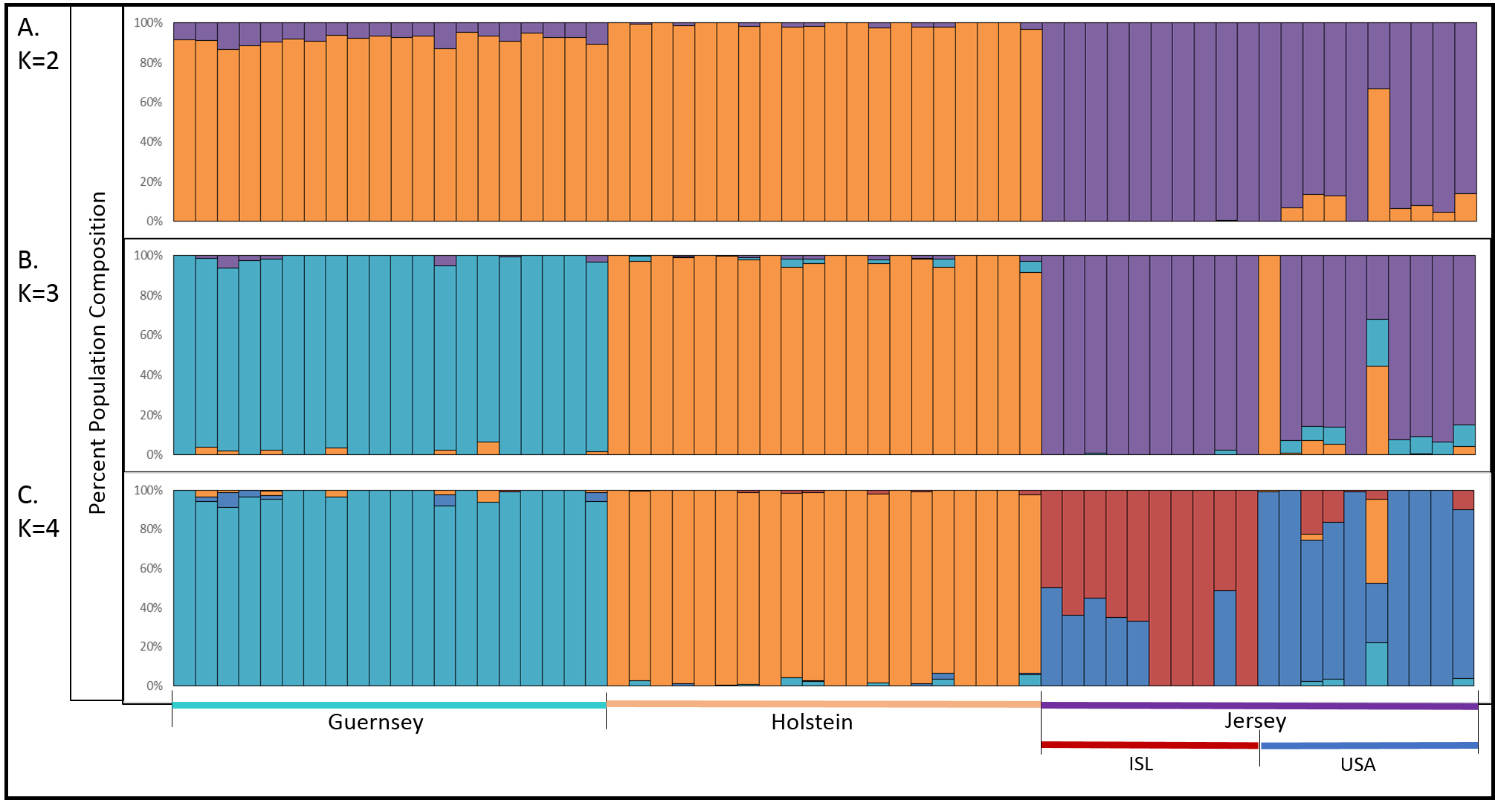


**Supplemental Figure 3.** Admixture analysis reflecting genetic clustering of breed using 20 individuals by breed; Holstein, Guernsey, and Jersey. Jersey is represented by 10 Island and 10 U.S. individuals. Individual vertical bars along the x-axis represent individual cattle which are grouped by breed. Genetic clusters corresponding to breed or population are denoted as follows: Guernsey (light/aqua blue), Holstein (orange), Jersey (purple), Island Jersey (ISL-red), and U.S. Jersey (USA-blue. The y-axis provides a measure of the percentage of each genetic population found within an individual. K represents the number of genetic populations used in each analysis with Figure A showing K = 2, Figure B showing K = 3, and Figure C showing K = 4.
